# Evidence-based rules from family practice to inform family practice; the learning healthcare system case study on urinary tract infections

**DOI:** 10.1186/s12875-015-0271-4

**Published:** 2015-05-16

**Authors:** Jean K Soler, Derek Corrigan, Przemyslaw Kazienko, Tomasz Kajdanowicz, Roxana Danger, Marcin Kulisiewicz, Brendan Delaney

**Affiliations:** Mediterranean Institute of Primary Care 19, Triq ir-Rand, Attard, Malta; Department of General Practice, HRB Centre for Primary Care Research, Beaux Lane House, Lower Mercer Street, Dublin, Ireland; Wroclaw University of Technology, Wybrzeze Wyspianskiego 27, 50-370 Wroclaw, Poland; Imperial College London, London, UK; Wolfson Chair of General Practice, King’s College London, Capital House, Guy’s Hospital, London, England

**Keywords:** Learning healthcare system, Data-mining, international classification of primary care, Diagnosis, Reason for encounter, Urinary tract infection, Pyelonephritis, Transform, Transition project, Electronic patient record

## Abstract

**Background:**

Analysis of encounter data relevant to the diagnostic process sourced from routine electronic medical record (EMR) databases represents a classic example of the concept of a *learning healthcare system (LHS)*. By collecting International Classification of Primary Care (ICPC) coded EMR data as part of the Transition Project from Dutch and Maltese databases (using the EMR TransHIS), data mining algorithms can empirically quantify the relationships of all presenting *reasons for encounter (RfEs)* and recorded diagnostic outcomes. We have specifically looked at new *episodes of care (EoC)* for two urinary system infections: simple urinary tract infection (UTI, ICPC code: U71) and pyelonephritis (ICPC code: U70).

**Methods:**

Participating family doctors (FDs) recorded details of all their patient contacts in an EoC structure using the ICPC, including RfEs presented by the patient, and the FDs’ diagnostic labels. The relationships between RfEs and episode titles were studied using probabilistic and data mining methods as part of the TRANSFoRm project.

**Results:**

The Dutch data indicated that the presence of RfE’s “Cystitis/Urinary Tract Infection”, “Dysuria”, “Fear of UTI”, “Urinary frequency/urgency”, “Haematuria”, “Urine symptom/complaint, other” are all strong, reliable, predictors for the diagnosis “Cystitis/Urinary Tract Infection” . The Maltese data indicated that the presence of RfE’s “Dysuria”, “Urinary frequency/urgency”, “Haematuria” are all strong, reliable, predictors for the diagnosis “Cystitis/Urinary Tract Infection”.

The Dutch data indicated that the presence of RfE’s “Flank/axilla symptom/complaint”, “Dysuria”, “Fever”, “Cystitis/Urinary Tract Infection”, “Abdominal pain/cramps general” are all strong, reliable, predictors for the diagnosis “Pyelonephritis” . The Maltese data set did not present any clinically and statistically significant predictors for pyelonephritis.

**Conclusions:**

We describe clinically and statistically significant diagnostic associations observed between UTIs and pyelonephritis presenting as a new problem in family practice, and all associated RfEs, and demonstrate that the significant diagnostic cues obtained are consistent with the literature. We conclude that it is possible to generate clinically meaningful diagnostic evidence from electronic sources of patient data.

**Electronic supplementary material:**

The online version of this article (doi:10.1186/s12875-015-0271-4) contains supplementary material, which is available to authorized users.

## Background

The process of diagnosis in family medicine (FM, synonymous with general practice) can be informed and enhanced by evidence emerging from data collected in routine clinical practice. The analysis of data on the elements of the encounter relevant to the diagnostic process sourced from routine electronic medical record (EMR) databases represents a classic example of the concept of a *learning healthcare system (LHS)*.

The International Classification of Primary Care (ICPC) acts as an ordering principle for FM data, allowing for direct international comparisons, and has the appropriate granularity for primary care data aggregation and analysis [1-5]. In the Transition Project, such ICPC data have been collected with EMRs in the Netherlands, Japan, Poland, Malta, Serbia, and other countries from the daily practice of a cohort of family doctors (FDs) using a similar methodology over time (one to eleven years) [4,6]. Such data have been used in the TRANSFoRm project to develop a diagnostic decision support system (DDSS) for FM, and the data have been recently published online as a repository of diagnostic association rules which are free to use to support family doctors’ (FDs) diagnostic processing [7,8].

The use of ICPC to study the epidemiology of FM has the advantage of allowing precise capture of *reason for encounter (RfE)* data, often ignored in FM research, and this allows further important perspectives into the process of diagnosis in FM [3,5,9-12].

This paper aims to exemplify the use of FM data to support diagnostic decisions in routine practice by analysing all possible associations between all the presenting *RfEs* in the Dutch and Maltese Transition Project databases (using the EMR TransHIS) and new *episodes of care (EoC)* for two urinary system infections: simple urinary tract infection (UTI, ICPC code: U71) and pyelonephritis (ICPC code: U70).

*The research question for this study is: “*What are the quantitative relationships between reasons for encounter and the diagnoses ‘UTI’ and ‘pyelonephritis’ (episode titles) within new episodes of care in routine family practice in practice populations from Malta and the Netherlands and how do these match the data already published in the literature?”.

## Methods

The public-domain EMR TransHIS, designed for use with ICPC, was used to collect data from participating FDs who recorded details of all their patient contacts in an *episode of care (EoC)* structure using ICPC. The study did not involve the collection of new data. Ethical approval was applied for locally, when appropriate, for individual studies based on these data in the Netherlands, Serbia and Malta. *Reasons for encounter (RfEs)* presented by the patient, all FD interventions, and the diagnostic labels recorded for each encounter were classified as recommended with ICPC (ICPC-2-E in Malta and Serbia, ICPC-1 in the Netherlands). All encounter data (face to face encounters in the office and at home, telephone consultations, repeat prescriptions, etc.) were analysed in an EoC structure to obtain complete data on incidence and prevalence, including patients presenting for a repeat prescription only [4-6,9-12].

An EoC is defined as a health problem from its first presentation by the patient to the FD, until the completion of the last encounter for it. It encompasses all contact elements related to that health problem. Its name (i.e. the diagnostic label of the EoC) may be modified over time, and in this article we refer to it as the *episode title* [2]*.* The last diagnosis made during an EoC is the current episode title. In this study, we focus on only two episode titles: urinary tract infection and pyelonephritis.

The RfE(s) is defined as an agreed statement of the reason(s) why a person enters the health care system, representing the demand for care by that person. The RfE should be recognized by the patient as an acceptable description of the demand for care [2]. FDs recording data for the Transition Project were trained to record RfEs according to the definitions above and the recommendations in the ICPC book, reflecting the patient’s symptoms and requests as expressed. Symptoms elicited during history-taking (i.e. the history of the presenting complaint) were recorded in a separate cell in the EMR TransHIS, but were not included in the analyses in this study [5,11].

The two databases each encompasses a defined time period: an average of 9,896 patients and 43,577 patient-years of observation over 5 years in Malta (2001–2005), and 15,318 patients and 158,370 patient-years over 11 years in the Netherlands (1995–2005). The practice populations in the Netherlands represent the registered patients, whilst the population in Malta represents the patients consulting over a five year period [5,9]. These databases are available in the public domain (www.transitieproject.nl; www.mipc.org.mt).

The relationships between RfEs and episode titles were studied using Bayesian probabilistic methods. According to Bayes’ Theorem, the post-test (posterior) odds of an event (i.e. a specific diagnosis being made) are equivalent to the pre-test odds multiplied by the *likelihood ratio* (*LR*) [5,10,11]. The LR values were calculated for a problem presenting for the first time at the beginning of a new EoC.

The LR is a mathematical, quantitative expression of the extent to which a symptom increases the probability of a given diagnosis. The positive LR (LR+) for the existence of the symptom is the odds that it will exist in a patient with the disease (relevant to diagnosis), in contrast to a patient without the disease. The negative LR (LR-) for absence of the symptom is the odds that the test will be negative in a patient with the disease, contrasted with a patient without the disease. We aggregated or pooled likelihood ratios across practices, as we have done in our previous studies [11,12].

It is possible to analyse such relationships between all possible combinations of episode titles and RfEs, using the TransHIS databases. Such an analysis has been performed and is presented on-line [8]. The website allows browsing, filtering, sorting and commenting of the results (association rules) of a data mining analysis platform that has been implemented to generate actionable clinical knowledge from electronic sources of coded primary care data. The user can filter the rules according to RfE, diagnostic cue (Anamnesis), diagnoses, sex and age groups as well as various quantitative measures. An open source data analysis tool, the Konstanz Information Miner (KNIME) has been used to define workflows that pre-process the TransHIS record data and derive association rules based on ICPC2 codes [13]. These rules identify all possible combinations of RfE, diagnostic cues and demographic variables (antecedent variables) that are linked with a recorded diagnostic outcome (consequent variable). The patient records loaded into KNIME consisted of only the first patient encounter relating to each new EoC for any patient. After cleaning (first encounter only from new episodes) 393,169 patient encounters were loaded into KNIME: 55,821 for Malta and 337,348 for the Netherlands. In total, 542,739 association rules were extracted from the data: 61,563 for Malta, 191,883 for the Netherlands, and 289,293 for both populations combined.

The distinct steps implemented in the data mining process required:

- derivation of association rules linking RfE, diagnostic cues and demographics to the recorded diagnoses made during the first encounter of a new episode of care
- calculation of association rule quality measures to determine the relative strength of each rule association derived, among others LR+, LR-
- filtering of association rules to allow selection of ‘high-quality’ association rules
- clinical review of selected rules to assess clinical validity of rules with respect to wider clinical body of evidence.

The analysis presented here was limited to two selected diagnoses, urinary tract infection (U71) and pyelonephritis (U70), for practical reasons. The minimum level of *clinical significance* for a LR was arbitrarily taken as representing a standardised difference of at least 0.10 (10%) [14]. Cut-off levels of >2 for the LR+ of a positive association, and <0.5 for the LR- of a negative association, were thus taken as minimum thresholds for clinical significance. LRs outside these limits were considered not clinically significant. On the other hand, LRs outside a second arbitrary threshold (LR+ >8, LR- <0.2) were considered to indicate a strong diagnostic association, and indicated as such in our conclusions [11,12]. Furthermore, as above, LRs which were not at least as large as their 95% confidence level (CI) were considered unreliable [11,12,14]. LRs based on cells with very small numbers were ignored. These criteria adjust for the increased chance of describing spurious associations due to the large number of repeated statistical tests in our analytic process, and also for errors in under-estimation of variance due to the effect of clustering [11,12,14].

## Results

The raw data output from the analysis applied using KNIME is provided in Additional file 1 and summarised in the form of tables in Additional files 2 and 3. These tables show the diagnostic associations identified for “UTI” and “Pyelonephritis” respectively. The positive likelihood ratios for all associated RfEs and the episode title “UTI” or “Pyelonephritis” in the two populations are listed. LRs are highlighted according to its value (clinical significance) and reliability (95% CI). Strong predictors (LR+ >8 or LR- <0.2, CI width smaller than or equal to the LR itself) are shown in red highlight. Weak predictors (LR+ >2-8, LR- 0.2-0.4, small CI) are shown in green highlight. LRs with a wide CI (larger than the observation itself) or which are not clinically significant (LR+ < =2, LR- > =0.5) or have a CI which includes unity are not highlighted.

The Dutch data and the combined dataset for “UTI” (Additional file 2) indicated that the presence of RfEs “Cystitis/Urinary Tract Infection”, “Dysuria”, “Fear of UTI”, “Urinary frequency/urgency”, “Haematuria”, “Urine symptom/complaint, other” are all strong and reliable predictors for the diagnosis “Cystitis/Urinary Tract Infection”. The RfEs “Incontinence urine,” “Urination problems, other”, “Abdominal pain localised, other”, “Flank/axilla symptom/complaint” are all reliable, but less strong predictors for the diagnosis “Cystitis/Urinary Tract Infection”. In the Dutch data the presence of RfEs “Vaginal symptom/complaint” or “Vaginal discharge” are strong but unreliable predictors to exclude a diagnosis of “Cystitis/Urinary Tract Infection”. The combined dataset indicated that “Vaginal symptom/complaint” was no longer a predictor for excluding a diagnosis of “Cystitis/Urinary Tract Infection”.

The Maltese data for “UTI” (Additional file 2) indicated that the presence of RfEs “Dysuria”, “Urinary frequency/urgency”, “Haematuria” are all strong, reliable, predictors for the diagnosis “Cystitis/Urinary Tract Infection”. The RfE “Abdominal pain localised, other” is a less strong but reliable predictor for the diagnosis “Cystitis/Urinary Tract Infection”.

In Additional file 3, the diagnostic associations for “Pyelonephritis” are analysed. The Dutch data indicated that the presence of RfEs “Flank/axilla symptom/complaint”, “Dysuria”, “Fever”, “Cystitis/Urinary Tract Infection”, “Abdominal pain/cramps general” are all strong, reliable, predictors for the diagnosis “Pyelonephritis”. The RfEs “Vomiting,” “Back symptom/complaint”, “Urinary frequency/urgency”, “Nausea”, “Abdominal pain localised, other”, “Low back symptom/complaint” are all less strong, but reliable predictors for the diagnosis “Pyelonephritis”. The combined dataset resulted in a number of weak predictors from the Dutch dataset becoming insignificant predictors. This loss of significance is due to the smaller number of cases of pyelonephritis combined from the Malta dataset.

The Maltese data set did not present any clinically and statistically significant predictors for pyelonephritis.

## Discussion

### Principal findings

This is a study of the clinical interpretation of two common symptom diagnoses, “Cystitis/Urinary Tract Infection” and “Pyelonephritis”, in routine family practice in two practice populations, Malta and the Netherlands as well as both combined. The data collected with ICPC were used to analyse the RfE associations between these two diagnoses made during the first encounter of an EoC starting with their presentation to the FD. A number of positive and negative diagnostic associations were found between these two RfEs and a number of episode titles. These associations were found to have different strengths of effect and differing precision of the effect estimate. However, a number of diagnostic associations were found to be similar across the two populations. A larger population would have given more precise LR estimates, and would likely have demonstrated even more congruence between these diagnostic associations.

### Implications of the findings

This study presents diagnostic associations from the perspective of the RfE, making it particularly useful to clinicians dealing with diagnostic challenges in the form of a newly presenting symptom in their daily practice. There were more similarities than differences in the diagnostic associations between RfEs and episode titles across populations, especially evidenced by the more frequent observations with narrower CIs.

### Comparisons with the literature

A key objective of this analysis was to compare for consistency of the clinical associations generated from our analysis with previous high quality studies of clinical evidence relating to the two diagnostic conditions. As such high quality evidence based reviews or guidelines of clinical evidence supporting Urinary conditions was chosen for comparison (Bent et al. 2002, SIGN UTI Guidelines 2012, European urology Guidelines 2013) [15-17]. A high level summary from the SIGN guidelines gives the following symptom based definitions of cystitis and pyelonephritis:

- UTI - “evidence of urinary tract infection with symptoms suggestive of cystitis (dysuria or frequency without fever, chills or back pain)”
- Pyelonephritis - “evidence of urinary tract infection with symptoms suggestive of pyelonephritis (loin pain, flank tenderness, fever, rigors or other manifestations of systemic inflammatory response)” [16].

The European Urology guidelines define cystitis symptoms as “Dysuria, frequency, urgency, pain or bladder tenderness”. These symptoms progress to pyelonephritis with additional symptoms of “Fever, Flank pain, Nausea, vomiting” [17].

The identified predictors from our analysis compare favourably with both the cystitis and pyelonephritis definitions. Our analysis indicated similar predictors in the form of urinary frequency, haematuria and dysuria from both population data sets for cystitis. Other weaker predictors are consistent including abdominal pain or flank pain. Predictors for pyelonephritis such as fever, flank/back pain, nausea and vomiting were also consistent with literature. In the Netherlands dataset, self-labelling by patients was also shown as a strong predictor for UTI. The presence of vaginal discharge was not quite strong enough to be considered a definitive excluding factor. Unlike the JAMA review, no association with fever was found for cystitis and this is consistent with later SIGN and European Urology guidelines which indicate this should be considered indicative of progression to pyelonephritis [15]. However we could not confirm any negative relationships between the presence of the symptoms “fever,” “chills” or “back pain” and a diagnosis of “Cystitis/Urinary Tract Infection.” Our analysis also highlighted cystitis itself as a significant predictor for pyelonephritis indicating the relationship and progression of these conditions into each other.

The JAMA review with quantified likelihood ratios for specific cues concluded that “specific combinations of symptoms (e.g. dysuria and frequency without vaginal discharge) raise the probability of UTI to more than 90%, effectively ruling in the diagnosis based on history alone” [15]. In our analysis dysuria with frequency was found to be the single biggest LR for a combination of cues and is consistent with the JAMA conclusions. Our calculated likelihood ratios were generally stronger than those from JAMA reviews which reflects firstly the larger volumes of data analysed in this study which did not pre-select patients with the index conditions, and secondly the effect of the lower prior probability with the earlier presentation of illness in primary care, as against emergency and secondary care (and consequently a higher positive LR). The clinical data published in the literature rarely include LRs based on primary care data, and further comparisons were therefore not possible, although desirable.

### Comparison between populations

The number of associations and their relative strengths were found to improve with analysis of larger volumes of data as shown by the relative comparison of generated associations from Netherlands and Malta. The smaller volume of Malta data tended to generated LRs that had wider confidence intervals. The prevalence of the condition has also shown to be important in requiring larger volumes of data as shown by the lack of predictors identified for the rarer pyelonephritis found in Malta data. The key cystitis indicators from Malta are consistent with the Netherlands data.

The relative lack of symptoms-oriented research into the diagnostic process in primary care makes finding comparable literature challenging. Most studies of diagnostic associations have been performed in datasets which are not exclusively or mainly from primary care. Additionally, most study a disease-label diagnosis and its associations with symptoms and test results as predictors, and not the other way around. In that sense, the diagnostic associations we have found may be more acceptable to and useful for clinicians. Furthermore, the congruency (and often statistical consistency) of diagnostic associations between these populations, and especially the fact that most of them are in the same direction from unity, sustain our confidence in their validity.

The results for the combined data (Malta and the Netherlands together) are heavily influenced by and agree with the Dutch data set as expected due to a larger number of patient encounters it contains. Where significant associations appear in the Dutch dataset without a comparable association in the smaller Malta dataset, this was reflected in some associations losing significance in the combined dataset (for example “vomiting” in the case of pyelonephritis).

### Limitations

This study examined associations between RfEs and episode titles at the *beginning* of a new EoC for that problem. It is quite possible that the diagnosis may have been revised over time during another consultation forming part of the EoC due to a change in the presentation, or a change in the diagnostic opinion of the FD, or consequent to the results of further testing, or through an opinion expressed by another health care provider, or otherwise.

A larger dataset would have quite likely picked up more significant associations, and provided more precise estimates of effects. The observed differences in diagnostic associations between populations may thus be due more to the lack of power to define the LRs more precisely, rather than due to any real difference in diagnostic processing of such RfEs.

We hereby publish the LRs used to study and describe these diagnostic associations in two different populations along with a combined dataset, and we offer our interpretation of the strength and reliability of such diagnostic associations, summarising the empirical data in text form. We understand that others may interpret these data differently, or may choose to accept different limits for the clinical and statistical significance of such associations.

### Strengths

This is a study of diagnostic associations for two common diagnoses in practice populations in very different health care settings, which has the advantage of empirical data collection and the validation of observations between two independent datasets. We analysed data on all RfEs presented and all diagnoses made in EoCs, which allows one to study any possible diagnostic association and define those which reach clinical and statistical significance. The presented data are but two examples. We also applied tight clinical and statistical significance limits to avoid describing spurious associations. The congruency of the diagnostic associations across populations sustains our confidence in their validity.

## Conclusions

The significant diagnostic cues obtained from the calculations performed on the Dutch data are consistent with the available clinical literature on LRs relating to both diagnostic conditions investigated. We conclude that it is possible to generate clinically meaningful diagnostic evidence from electronic sources of patient data.

Further research in this area is important to sustain the development of FM as a clinical and academic discipline, and to inform decision support tools and systems developed for family practice. The assumptions we have made on the clinical and statistical significance limits for a diagnostic association, and the method we have used to interpret and summarise such diagnostic associations in different populations, are presented to the scientific community for discussion.

### Availability of supporting data

All supporting data is provided as an additional file showing the raw excel output from the KNIME data mining process upon which Additional files 2 and 3 were prepared.

## Additional files

Additional file 1:
**Raw exported data from KNIME data mining tool used to create** **Table S1** **and** **Table S2.**
Additional file 2: Table S3. Showing positive likelihood ratios for associated RfEs (label and ICPC code listed) and the episode title “UTI” in two populations. Additional file 3: Table S4. Showing positive likelihood ratios for associated RfEs (label and ICPC code listed) and the episode title “pyelonephritis” in two populations.

## Abbreviations

DDSS: Diagnostic Decision Support
EMR: Electronic Medical Record
EoC: Episode of Care
ICPC: International Classification of Primary Care
KNIME: Konstanz Information Miner
RfE: Reason for Encounter
