# Supplementary material for: Evidence-based rules from family practice to inform family practice; the learning healthcare system case study on urinary tract infections
Source: BMC Fam Pract. 2015 May 16;16:63. doi: 10.1186/s12875-015-0271-4 (PMC4438341; doi:10.1186/s12875-015-0271-4)
Supplement: Additional file 2: Table S3. — Showing positive likelihood ratios for associated RfEs (label and ICPC code listed) and the episode title “UTI” in two populations. [file 12875_2015_271_MOESM2_ESM.pdf]

Table 1 Positive likelihood ratios for associated RfEs (label and ICPC code listed) and the episode title “UTI” in two populations

RfE Code	RfE Label	LR+ Netherlands	LR+ Malta	LR+ Combined	LR+ JAMA Review
U04	Incontinence urine	6.27 (5.04 – 7.80)	38.94 (15.93 – 95.2)	7.09 (5.74 – 8.76)	
U07	Urine symptom/complaint, other	16.34 (11.51 – 23.21)	50.07 (21.99 – 114.01)	18.75 (13.59 – 25.87)	
U05	Urination problems, other	6.35 (4.81 – 8.4)		6.85 (5.25 – 8.95)	
L05	Flank/axilla symptom/complaint	2.08 (1.58 – 2.75)		2.23 (1.70 – 2.93)	1.1 (0.9 – 1.4)
U06	Haematuria	22.40 (18.63 – 26.94)	74.57 (49.54 – 112.26)	26.23 (22.19 – 31.01)	2.0 (1.3 – 2.9)
D06	Abdominal pain localized, other	2.59 (2.33 – 2.88)	7.67 (6.56 – 8.96)	3.09 (2.82 – 3.38)	1.1 (0.9 – 1.4)
U95	Urinary calculus	3.15 (1.38 – 7.22)		3.09 (1.35 – 7.05)	
U29	Urinary symptom/complaint, other	17.16 (10.8 – 27.29)		18.17 (11.44 – 28.86)	
U27	Fear of urinary disease, other	44.86 (38.7 – 52.02)		46.18 (39.89 – 53.45)	
U01	Dysuria/painful urination	84.02 (77.87 – 90.67)	216.48 (180.68 – 259.39)	94.08 (87.70 – 100.93)	1.5 (1.2 – 2.0)
U71	Cystitis/urinary infection, other	185.80 (165.50 – 208.58)	305.98 (85.56 – 1094.27)	199.55 (177.81 – 223.95)	4.0 (2.9 – 5.5)
U02	Urinary frequency/urgency	44.65 (41.59 – 47.94)	151.66 (122.41 – 187.9)	50.78 (47.48 – 54.31)	1.8 (1.1 – 3.0)
A02	Chills	2.68 (1.37 – 5.25)		2.31 (1.35 – 3.96)	
U13	Bladder symptom/complaint, other	36.49 (22.41 – 59.68)		39.24 (24.11 – 63.88)	
U14	Kidney symptom/complaint	6.08 (2.59 – 14.3)		6.10 (2.61 – 14.30)	
X15	Vaginal symptom/complaint, other	0.48 (0.26 – 0.9)	2.65 (1.18 – 5.95)	0.68 (0.42 – 1.12)	
A03	Fever	0.81 (0.68 – 0.94)		0.72 (0.63 -0.82)	1.6 (1.0 – 2.6)
X14	Vaginal Discharge	0.13 (0.06 – 0.32)	2.12 (0.86 – 5.13)	0.25 (0.14 – 0.47)	
U01 and U02	Dysuria with Urinary Frequency	193.87 (165.54 – 227.05)	745.08 (431.150 – 1287.6)	222.67 (191.39 – 259.08)	

LRs are highlighted according to the value (clinical significance) and reliability (95% CI). Strong predictors (LR+ >8 or LR- <0.2, CI width being equal to or smaller than the size of the observation itself) are in red. Weak predictors (LR+ >2-8, LR- 0.2-0.4, small CI) are in green. Associations with a wide CI (larger than the observation itself) or which are not clinically significant (LR+ < =2, LR- > =0.5) or have a CI which includes unity are not included.
