# Supplementary material for: Evidence-based rules from family practice to inform family practice; the learning healthcare system case study on urinary tract infections
Source: BMC Fam Pract. 2015 May 16;16:63. doi: 10.1186/s12875-015-0271-4 (PMC4438341; doi:10.1186/s12875-015-0271-4)
Supplement: Additional file 3: Table S4. — Showing positive likelihood ratios for associated RfEs (label and ICPC code listed) and the episode title “pyelonephritis” in two populations. [file 12875_2015_271_MOESM3_ESM.pdf]

Table 2 Positive likelihood ratios for associated RfEs (label and ICPC code listed) and the episode title "pyelonephritis" in two populations

RfE Code	RfE Label	LR+ Netherlands	LR+ Malta	LR+ Combined
A02	Chills	57.97(25.88 – 129.82)		33.49 (15.06 – 74.45)
U02	Urinary frequency/ urgency	7.17 (4.31 – 11.94)		7.24 (4.34 – 12.05)
U70	Pyelonephritis / pyelitis	1001 (494.12 – 2027.87)		1001 (493.93 – 2028.62)
U06	Haematuria	17.38 (7.85 – 38.48)		16.57 (7.49 – 36.68)
U27	Fear of urinary disease, other	18.75 (9.84 – 35.75)		20.63 (10.82 – 39.34)
L05	Flank/axilla symptom/ complaint	55.16(41.65 – 73.06)		59.60 (44.97 – 78.99)
A03	Fever	11.81(9.88– 14.3)	4.19(1.71 – 10.25)	9.00 (7.51 – 10.78)
U71	Cystitis/urinary infection, other	11.70(7.15 – 19.13)		13.09 (8.00 – 21.42)
D01	Abdominal pain/cramps general	9.61(5.98 – 15.46)		7.03 (4.38 – 11.31)
L02	Back symptom/ complaint	7.41(4.45 – 12.33)	17.44(7.12 – 42.75)	7.00 (4.42 – 11.08)
D10	Vomiting	7.77(4.58 – 13.21)		4.95 (2.91 – 8.41)
D09	Nausea	6.79(4 – 11.54)	5.94(0.95 – 37.17)	5.75 (3.45 – 9.57)
U01	Dysuria/painful urination	14.07 (9.85 – 20.11)	28.25(8.48 – 94.1)	14.42 (10.22 – 20.33)
D06	Abdominal pain localized, other	6.14 (4.27 – 8.84)	21.19(12.36 – 36.33)	6.48 (4.66 – 9.01)
L03	Low back symptom/ complaint	2.33(1.34 – 4.04)		2.50 (1.44 – 4.34)
A06	Fainting/ synchope		28.83(4.59 – 181.11)	0.98 (0.14 – 6.94)

LRs are highlighted according to the value (clinical significance) and reliability (95% CI). Strong predictors (LR+ >8 or LR- <0.2, CI width being equal to or smaller than the size of the observation itself) are in red. Weak predictors (LR+ >2-8, LR- 0.2-0.4, small CI) are in green. Associations with a wide CI (larger than the observation itself) or which are not clinically significant (LR+ < =2, LR- > =0.5) or have a CI which includes unity are not included.
